# Supplementary figures and images for: Improving therapeutic protein secretion in the probiotic yeast Saccharomyces boulardii using a multifactorial engineering approach
Source: Microb Cell Fact. 2023 Jun 7;22:109. doi: 10.1186/s12934-023-02117-y (PMC10245609; doi:10.1186/s12934-023-02117-y)

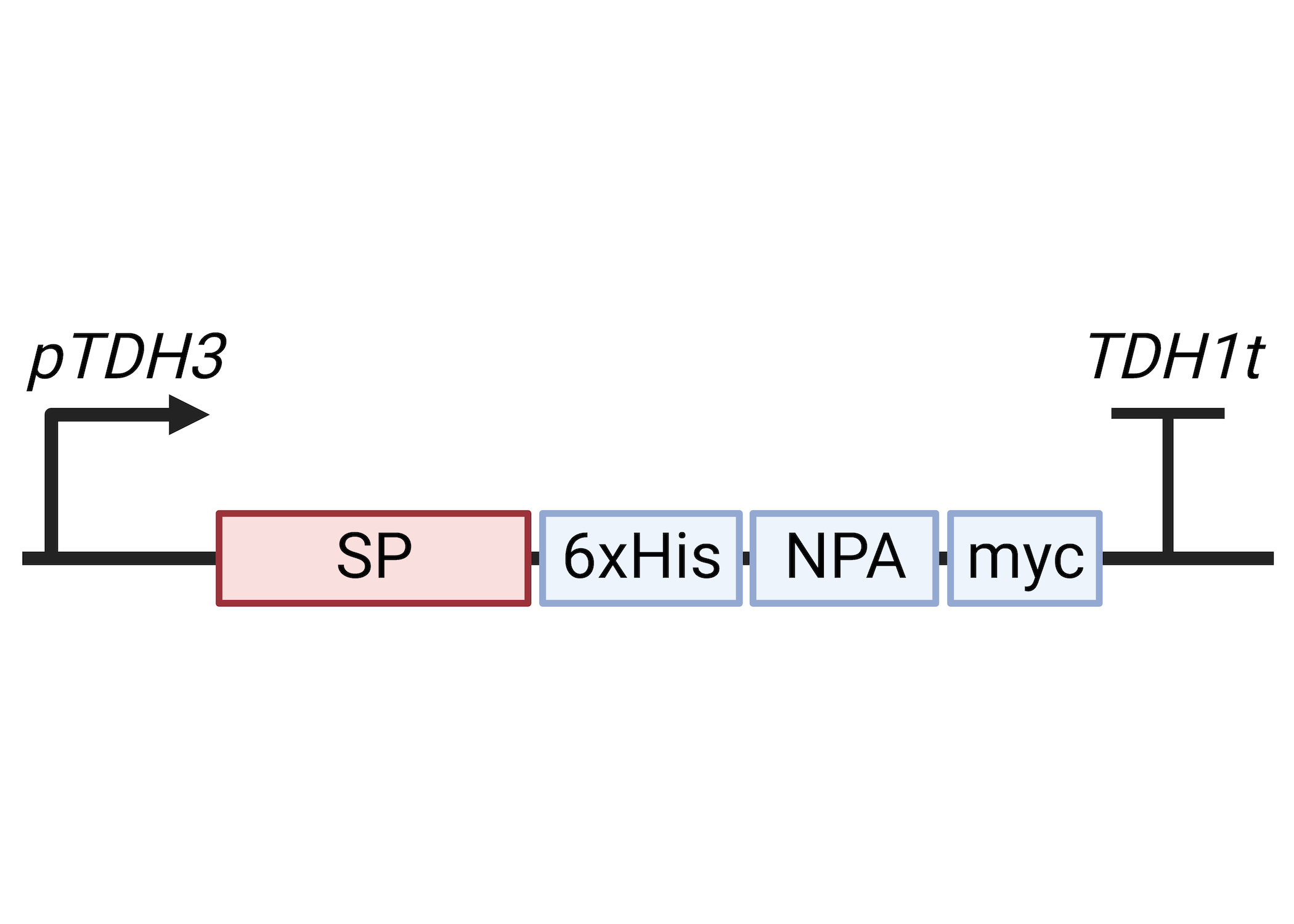

Supplement: Supplementary file 1 — Additional file 1: Figure S1. Schematic overview of the expression cassette for secretory production of therapeutic peptide NPA. The expression was regulated by the strong constitutive TDH3 promoter and TDH1 terminator. Native and synthetic signal peptide sequences were cloned upstream of the NPA sequence, which was flanked by a poly-histidine tag and myc tag on its N- and C-terminus, respectively. This cassette was inserted into high- or low-copy plasmids or inserted into the genome. [file 12934_2023_2117_MOESM1_ESM.jpg]

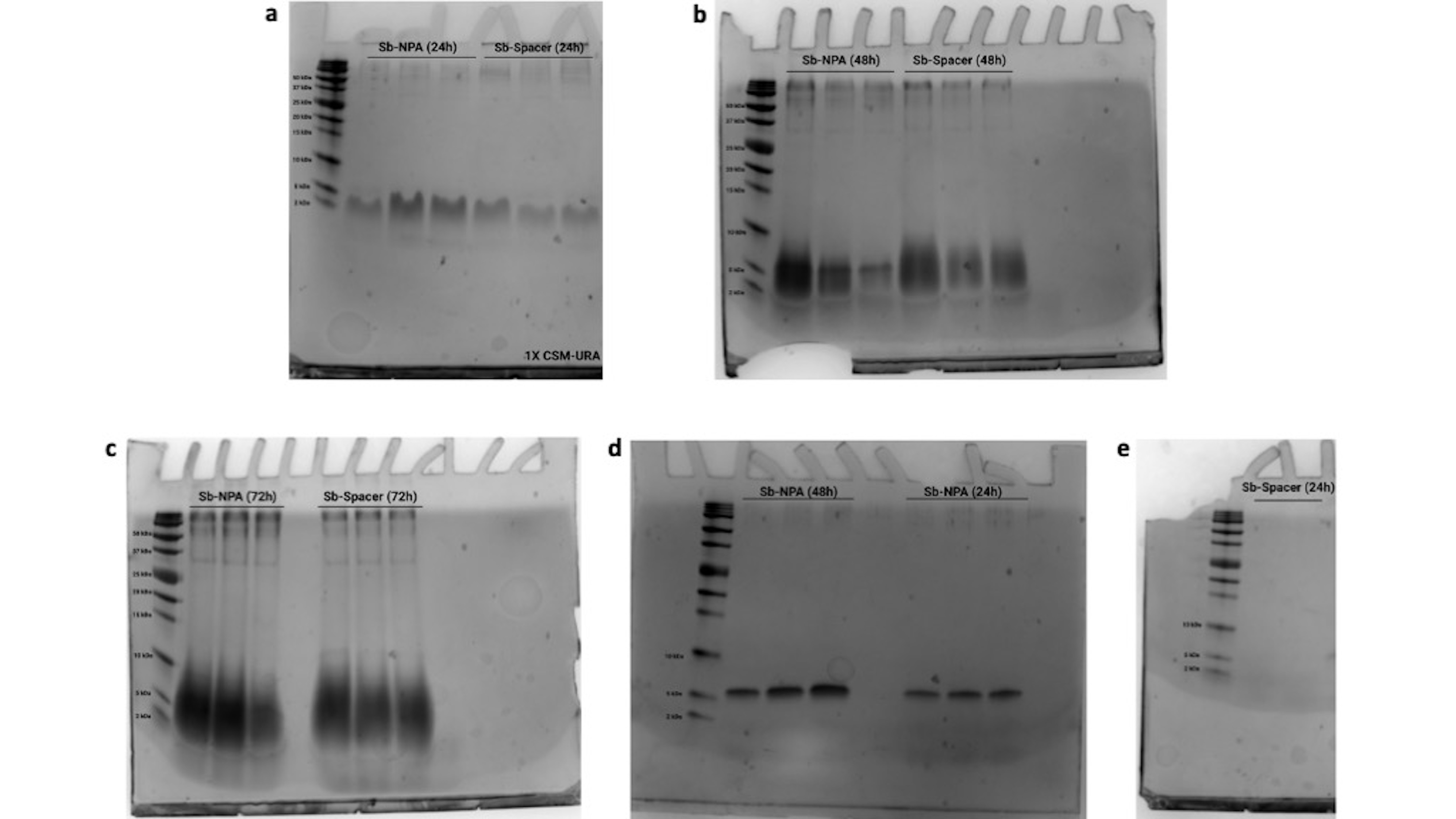

Supplement: Supplementary file 2 — Additional file 2: Figure S2. Detection of NPA secreted in culture media via SDS-PAGE. a Sb-NPA was cultured inCSM-URA for 24 hours and supernatants were precipitated and run on 4-20% Tris-Tricine gels. Sb-NPA and Sb-Spacer supernatants were collected after 48 hoursand 72 hours, precipitated, and run on 4-20% Tris-Tricine gels. Each strain was cultured in triplicate in culture tubes at 37 °C. Precipitated Sb-NPAand Sb-Spacersupernatants from 24 hoursand 48 hourswere processed through Dynabeads His-Tag Isolation and Pulldown to isolate poly-histidine tagged NPA present in the precipitates. Eluates were run on 4-20% Tris-Tricine gels. [file 12934_2023_2117_MOESM2_ESM.jpg]

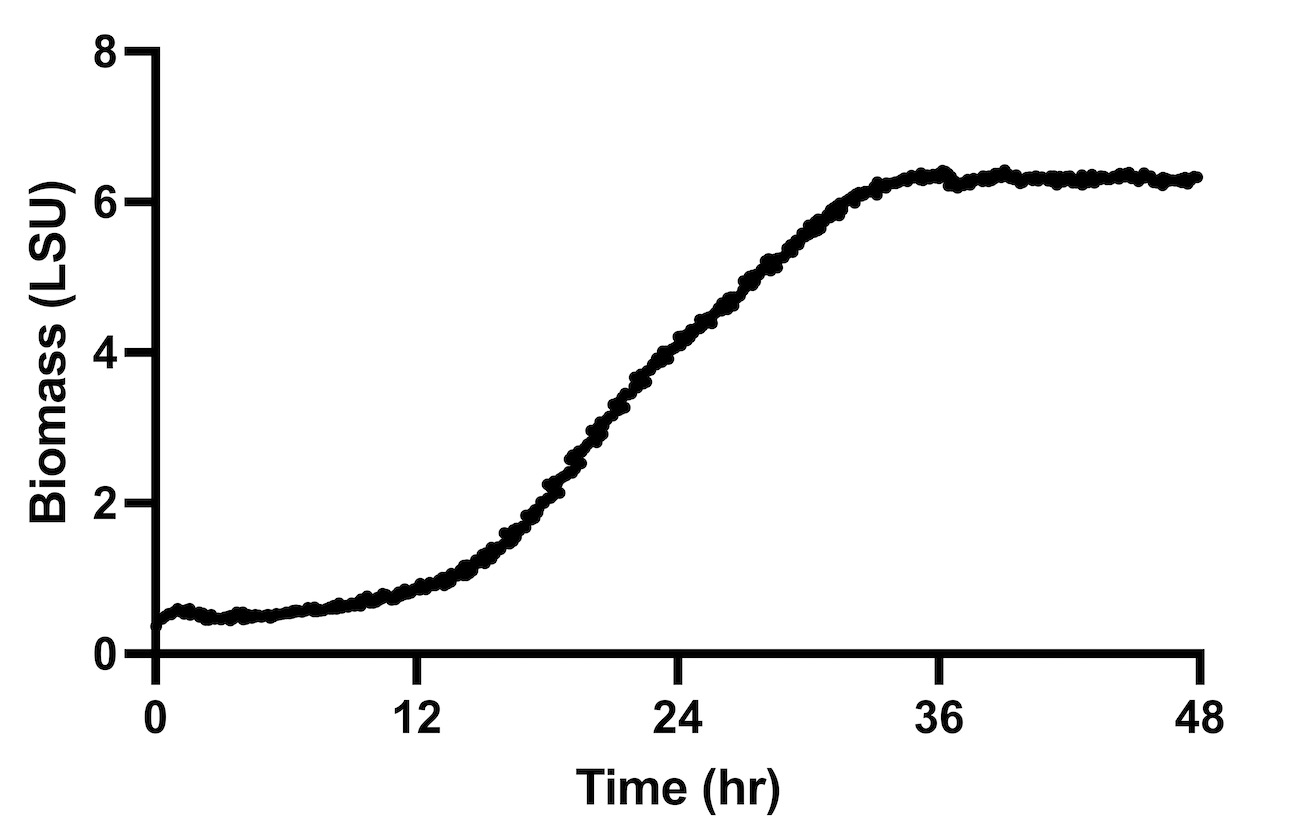

Supplement: Supplementary file 3 — Additional file 3: Figure S3. Growth profile of Sb-NPA. Sb NPA was cultured for 48 hours in microfermentor inCSM-URA media. Biomass was collected as light scattering units. The average LSU values for the triplicates for each timepoint were plotted. [file 12934_2023_2117_MOESM3_ESM.jpg]

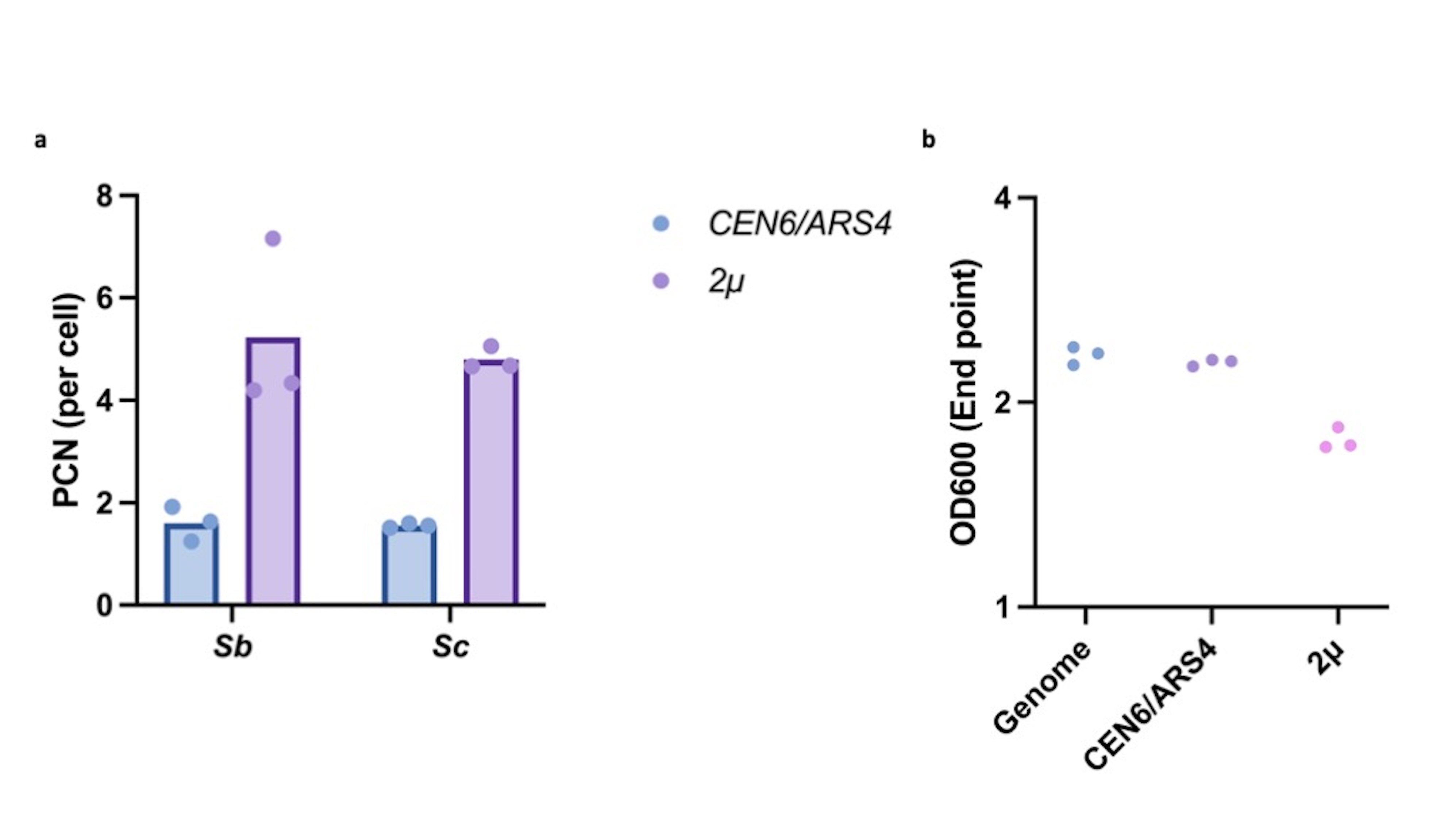

Supplement: Supplementary file 4 — Additional file 4: Figure S4. Plasmid copy numbers and final cell density of NPA secretor strains with varying copy numbers. a Each strain was cultured in triplicates in culture tubes for 24 hours at 37 ℃. Total DNA was isolated and PCN is determined by qPCR. Dots represent PCN in each cultivation for a given strain. b Each strain was cultured in triplicates in FlowerPlates in Biolector II for 48 hours at 37 ℃. OD600 values were obtained at the end of the cultivations via spectrophotometer. Dots represent the OD600 values in each cultivation for a given strain. [file 12934_2023_2117_MOESM4_ESM.jpg]

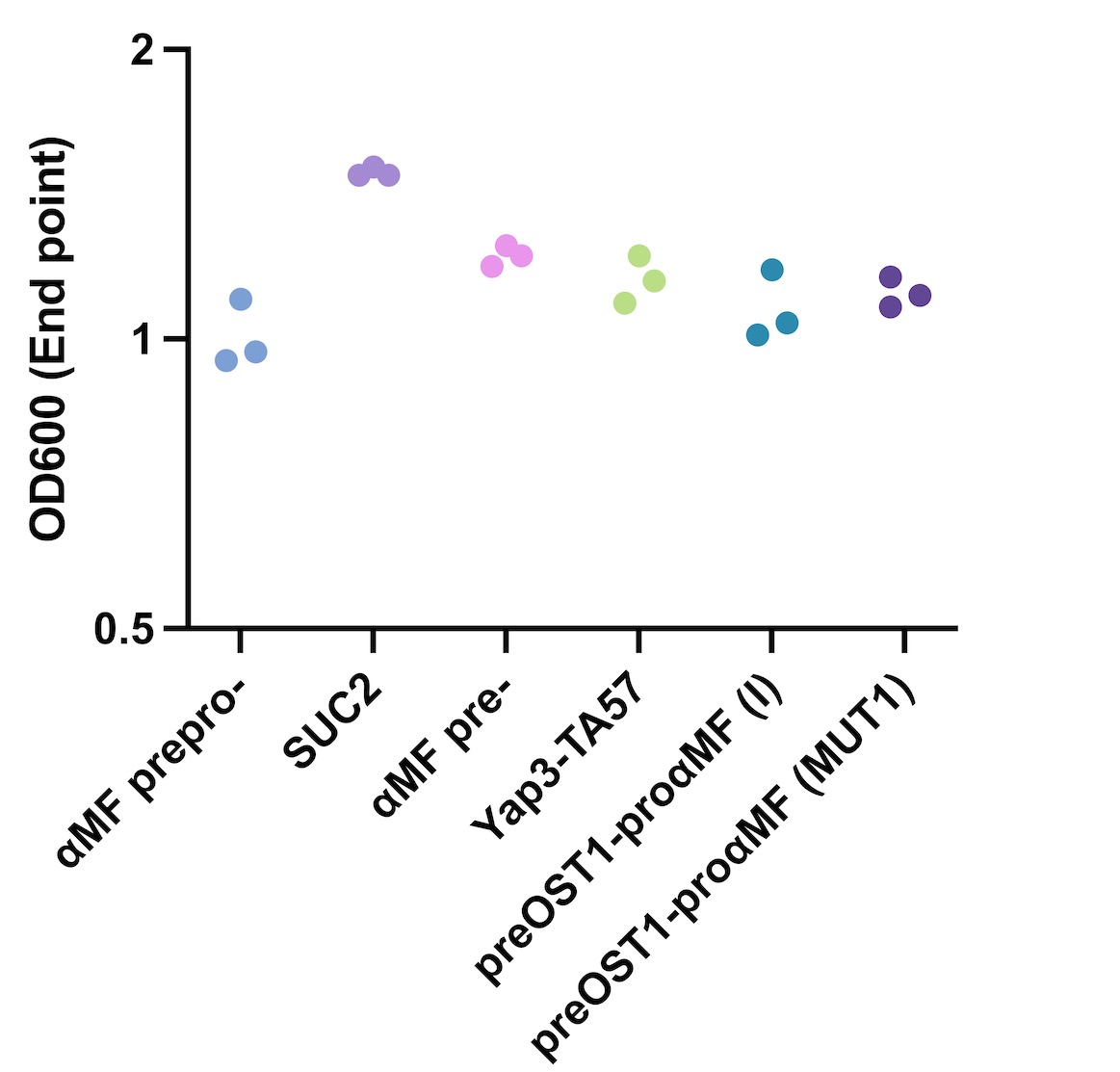

Supplement: Supplementary file 5 — Additional file 5: Figure S5. Final cell density of NPA secretor strains with varying secretion signals. NPA secretion cassettes with 3 native and 3 synthetic secretion signals were cloned into a backbone with a high-copyorigin. Each strain was cultured in triplicate in FlowerPlates in a Biolector II for 36 hours at 37 °C. Bars represent the average endpoint cell density across three cultivations, and dots represent the endpoint cell density in each cultivation. [file 12934_2023_2117_MOESM5_ESM.jpg]

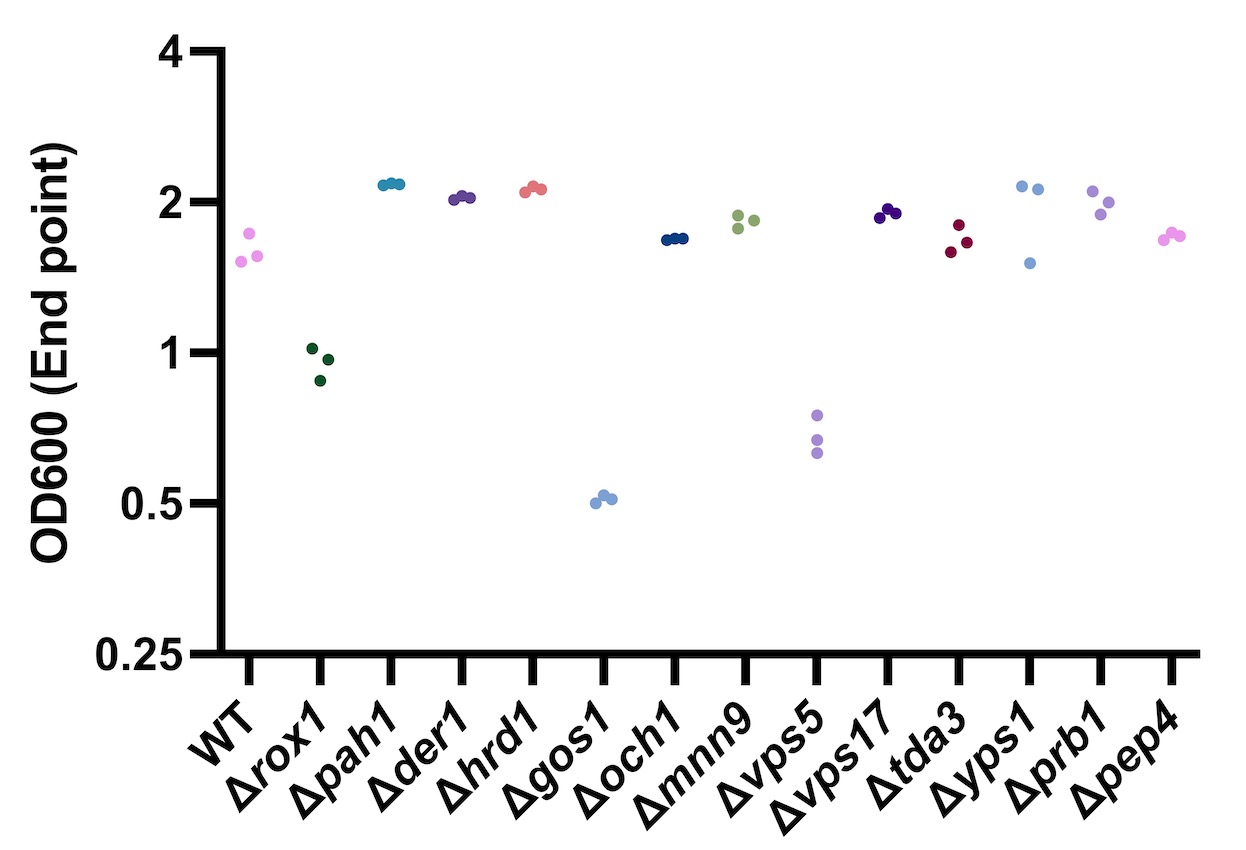

Supplement: Supplementary file 6 — Additional file 6: Figure S6. Final cell density of NPA secretor strains with varying secretory gene deletions. Each strain was cultured in triplicates in FlowerPlates in a Biolector II for 48 hours at 37 °C. Each dot represents the endpoint OD600 for each cultivation. [file 12934_2023_2117_MOESM6_ESM.jpg]

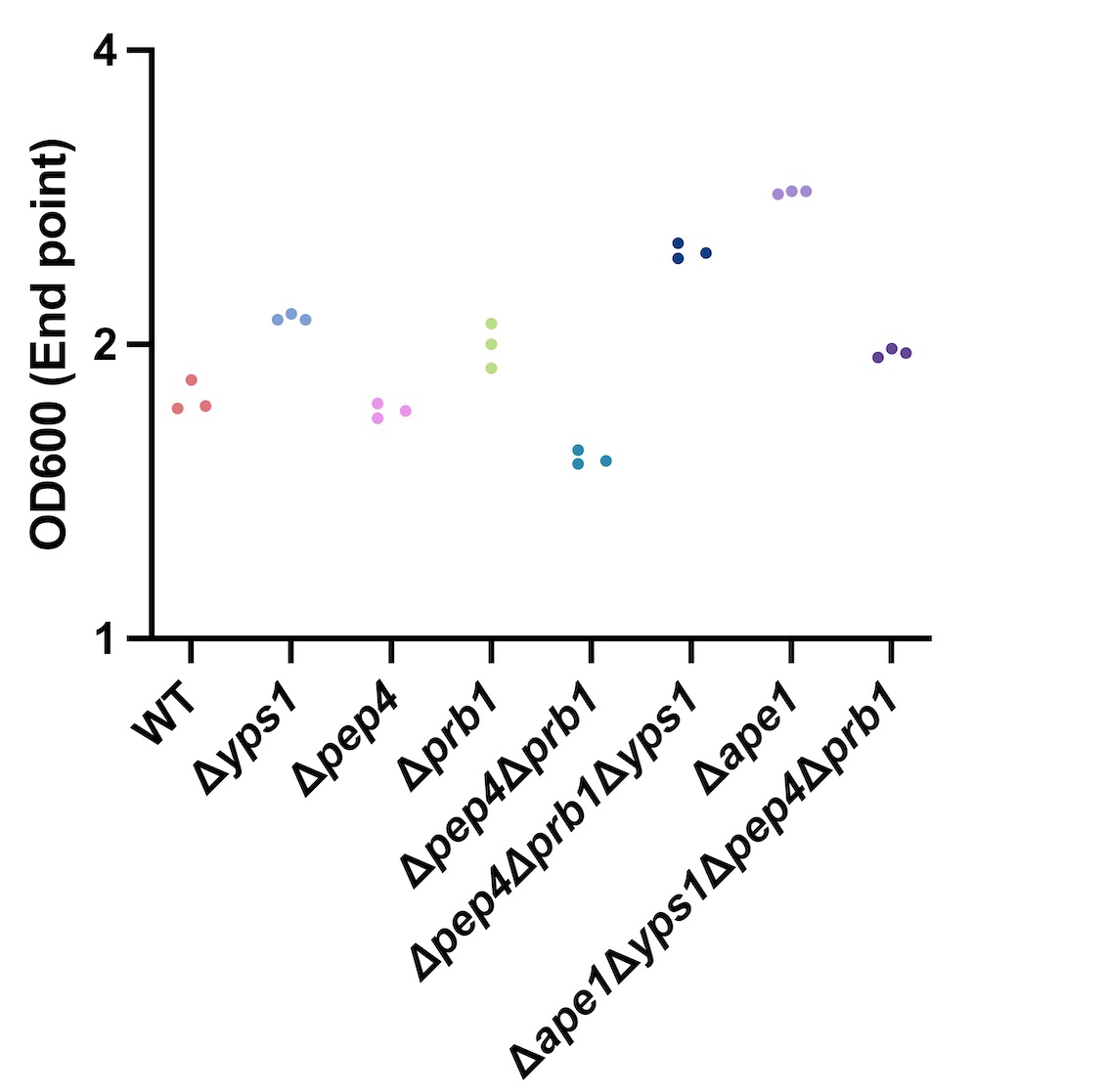

Supplement: Supplementary file 7 — Additional file 7: Figure S7. Final cell density of NPA secretor strains with combinatorial secretory gene deletions. Each strain was cultured in triplicates in FlowerPlates in a Biolector II for 48 hours at 37 °C. OD600 values were obtained at the end of the cultivation via a spectrophotometer. Dots represent the OD600 values in each cultivation for a given strain. [file 12934_2023_2117_MOESM7_ESM.jpg]

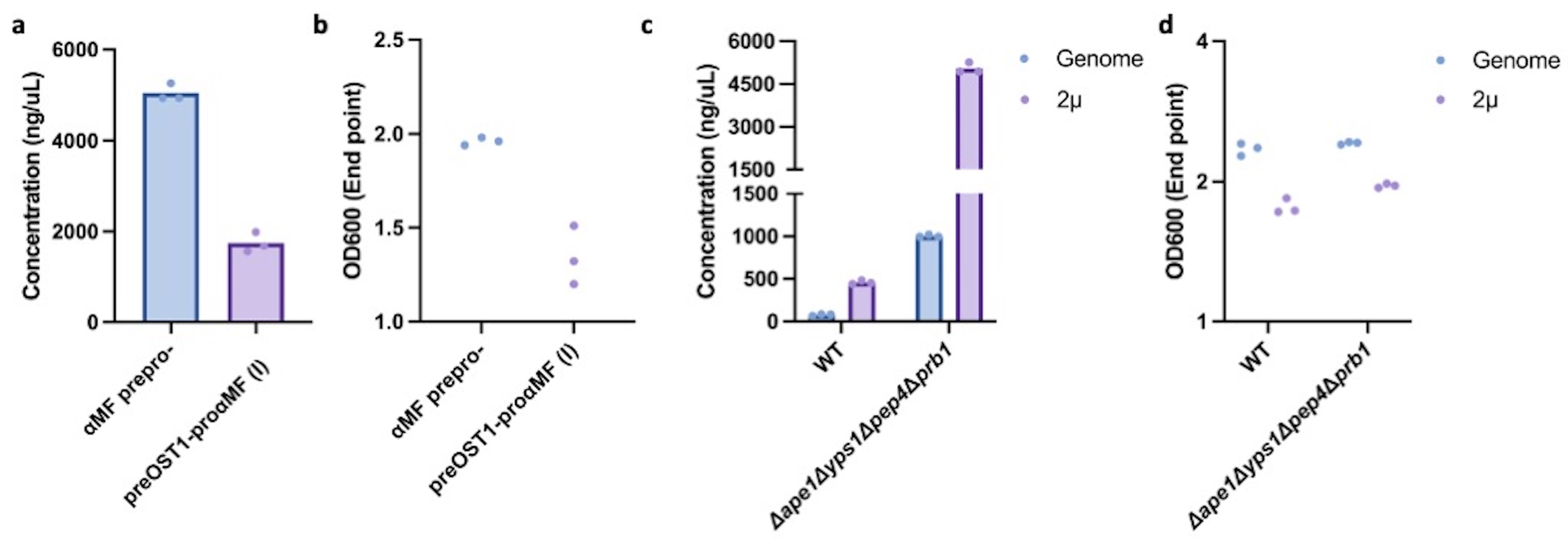

Supplement: Supplementary file 8 — Additional file 8: Figure S8. Effect of secretion signal and copy number on NPA secretion and final cell density in the quadruple knockout Sb strain. a NPA concentration in supernatant in the quadruple knockout Sb strain expressing NPA via the αMF secretion signalor preOST1-proαMFsecretion signal. b Final cell densityfor cultures in panel. c NPA concentration in culture supernatant in wild-type and quadruple knockout Sb strains expressing NPA via the αMF secretion signal integrated into INT1 site on the Sb genomeor cloned into a high-copyyeast plasmid. d Final cell densityfor cultures in panel c. Each strain was cultured in triplicates in FlowerPlates in a Biolector II for 48 hours at 37 °C. OD600 values were obtained at the end of the cultivation via a spectrophotometer. For NPA concentration plots, bars represent the average NPA concentration across three cultivations and dots represent the NPA concentration in each cultivation for a given strain. For OD600 plots, dots represent the OD600 values in each cultivation for a given strain. [file 12934_2023_2117_MOESM8_ESM.jpg]
